# Systematic review of cost projections of new vaccine introduction

**DOI:** 10.1016/j.vaccine.2024.01.024

**Published:** 2024-02-15

**Authors:** Ann Levin, Karene Hoi Ting Yeung, Raymond Hutubessy

**Affiliations:** a Levin & Morgan LLC, Bethesda, Maryland, United States; b Department of Immunization, Vaccines and Biologicals, World Health Organization, 20, Avenue Appia, 1211 Geneva 27, Switzerland

**Keywords:** Systematic review, Cost projection, New vaccine introduction, Vaccine delivery cost, Immunization

## Abstract

**Background:**

A recent review of guidance documents on vaccine delivery costing revealed current guidance on cost projections for new vaccine introduction has gaps on methods of sampling, data collection and analysis. In preparation for updating the respective guidance, this systematic review was undertaken to qualitatively assess methodologies used in new vaccine cost projection studies. This will inform researchers and stakeholders about the methods of new vaccine introduction cost projections for strategic directions in countries where cost data are not available.

**Methods:**

We systematically searched four search engines (PubMed, Cochrane Open Access, Mendeley and Google Scholar) for articles on cost projections for new vaccines published between 1999 and 15 June 2022. We developed inclusion and exclusion criteria for the selection of articles and analyzed the results using a PRISMA 2020 flow diagram.

**Results:**

Out of 1,108 articles identified, 171 met the criteria for inclusion in the study. Half of the articles were from high-income countries (50%), and most cost projections were part of cost-effectiveness analysis (84%). The most common source of cost data was secondary national information (43%), followed by author’s assumptions (17%), secondary international information (14%), and primary data collection (7%). 19% of studies didn’t include costs to deliver vaccines in their cost estimation. Among studies that included secondary vaccine delivery costs, approximately half only calculated vaccine administration costs (50%), while 35% included incremental system costs and 15% utilized ingredients data. Two thirds of the studies were conducted to inform policymakers of the cost-effectiveness or cost-benefit of introducing the vaccine.

**Conclusions:**

Half of the economic evaluations on new vaccine introductions only included partial vaccine delivery costs. Thus, total costs of vaccine introduction were often being underestimated in economic evaluations. This suggests that guidelines on economic evaluations and journals should recommend that authors include more extensive vaccine delivery costs in their studies.

## Highlights

• The majority of new vaccine cost projections have been conducted in high- and middle-income countries.
• Most cost projections were part of a cost-effectiveness or cost-benefit analysis to assist decision-making on introducting a new vaccine.
• Over half of the studies utilized secondary cost data, indicating the importance of improving accuracy of vaccine cost projection estimates.
• Around half of studies underestimated delivery costs of introducing new vaccines since these left out introduction and operational costs.

## 1 Background

Vaccination is one of the most effective interventions against infectious diseases. Policymakers need various information such as efficacy, effectiveness and implementation costs in their decision-making on vaccine introduction at country-level and in strategic planning for resource mobilization for global initiatives like the Immunization Agenda 2030 [1]. Cost information is important for strategic planning for development of budgets as well as necessary for evaluating cost-effectiveness of a new vaccine for priority-setting related questions. It also assists policymakers and program managers in assessing the affordability and sustainability through budget impact assessments of introducing the new vaccine in their decision-making. Cost projections will also help preparedness and response investments to inform optimal surge financing needs for the next pandemic [2]. Cost projections of new vaccine introduction estimate future costs through projection of the value of recurrent and capital inputs needed for national immunization programs, typically using incremental costing [3].

A recent review [3], [4] of guidance documents on vaccine delivery costing revealed that current guidance on cost projection has some gaps on methods of sampling, data collection and analysis [5]. Therefore, the Immunization and Vaccine-related Implementation Research Advisory Committee (IVIR-AC) recommended that the World Health Organization (WHO) consider updating a WHO guidance document [6] on estimating costs of introducing new vaccines into the national immunization system to reflect the latest methods of projecting vaccine delivery costs. In addition, since there are no existing reviews of cost projections of new vaccine introduction, they suggested that a systematic review be conducted to fill a key evidence gap on methods that new vaccine costing studies used to project vaccine delivery costs. The findings of this systematic review will inform researchers and stakeholders about the methods used in cost projections of new vaccine introduction for strategic directions in countries where cost data are not available, e.g., low-income countries, as well as feed into the development of guidelines for new vaccine cost projections.

The objective of this review is to conduct a systematic review of methods used to estimate delivery costs of new vaccine introduction in peer-reviewed literature, specifically the following: (i) objectives; (ii) data collection and sampling methods; (iii) data sources used; (iv) methods of analyses; (v) characterization of data uncertainty; and (vi) use cases of cost estimates.

## 2 Methods

### 2.1 Literature search strategy

We systematically searched four search engines to locate articles with cost projections for new vaccine introduction on June 15–16, 2022: PubMed, Cochrane Open Access, Mendeley, and Google Scholar.^1^ Search terms included:

• Vaccines: coronavirus disease 2019 (COVID-19), diphtheria- pertussis-tetanus (DPT), haemophilus influenzae type b (Hib), hepatitis B, human papillomavirus (HPV), seasonal influenza, measles-rubella, meningococcal, oral cholera, pneumococcal (PCV), polio, rotavirus, RTS,S, typhoid conjugate, varicella, yellow fever
• Other terms: vaccine cost, cost projection, costing, vaccine, vaccination, delivery costs, immunization

The full search strategy has been described in Appendix 1 and the electronic search strategy for PubMed has been provided as an example of the search in the electronic database.

### 2.2 Study selection

Studies were considered eligible if they met the following inclusion criteria: (i) included cost projections of vaccine introductions, (ii) were specific to one or less than ten countries, and (iii) were published from 1990 until June 15, 2022. Studies were excluded if they had the following: (i) were retrospective costing, (ii) estimated global or regional costs, (iii) were written in languages other than English, or (iv) were published before 1990.

The inclusion and exclusion criteria were applied to all stages of review: title screening, abstract review and full-text review. Articles were initially screened from the titles of all the citations retrieved from the electronic databases. Abstract review of publications was then followed by full-text review. Articles that met the inclusion criteria and did not meet the exclusion criteria were included in the review. If the fulfilment of all inclusion criteria could not be determined through title screening or abstract review, as long as not meeting any exclusion criteria, the articles were included for the next screening stage. Additional relevant articles were obtained from the reference lists of the included reviews and individual studies. We did not include gray literature due to concerns about the quality of the review process for gray literature, its accessibility and potential biases [7], [8], [9].

### 2.3 Data extraction, analyses and synthesis

A matrix of vaccines was developed to record the number of articles at each stage of screening. Data were extracted and entered into an Excel workbook with one worksheet for each vaccine. Extracted data include article titles, authors, years of publication, journal where published, study objectives, vaccine costs, delivery costs and their calculation, citations for delivery cost, data collection and sampling methods, methods of analyses including perspective and discount rates, methods to account for uncertainty, and use cases of cost estimates. The data collected were analyzed and synthesized and displayed in tables, graphs and charts.

## 3 Results

### 3.1 Study selection

**Fig. 1:**
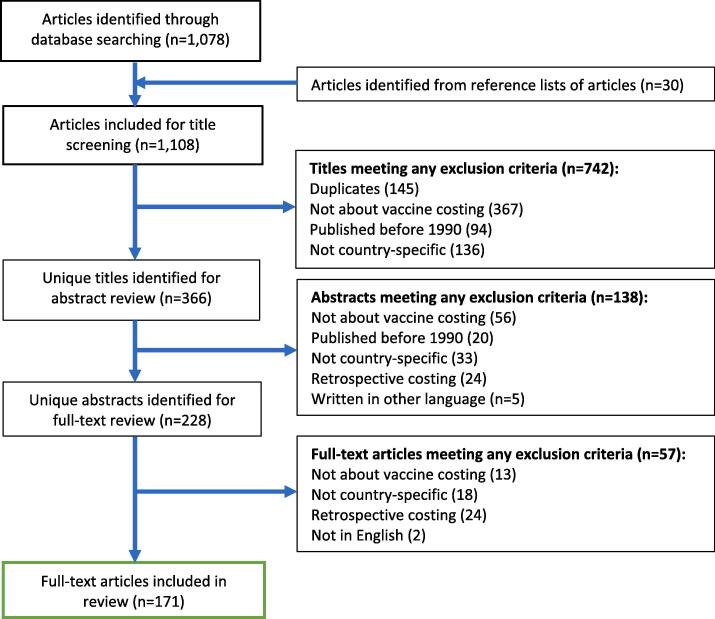
PRISMA flow diagram of study selection for the systematic review.

1,078 articles were identified from the four databases – 592 from PubMed, 86 from Cochrane, 132 from Mendeley, and 268 from Google Scholar. An additional 30 articles were obtained from reference lists of included articles. Of the total 1,108 articles, 366 were eligible for abstract screening after removing 742 articles that were either duplicates, not cost-related, published before 1990, or not country-specific during title review. An additional 138 articles were excluded after abstract review. Some 228 articles were identified for full-text review and 57 of these did not meet the inclusion criteria. Finally, 171 articles were included for analysis (Appendix 2). Fig. 1 is a PRISMA flow diagram [10] and presents the number of studies that met the criteria for inclusion and exclusion during the review process.

### 3.2 Characteristics of studies

**Fig. 2:**
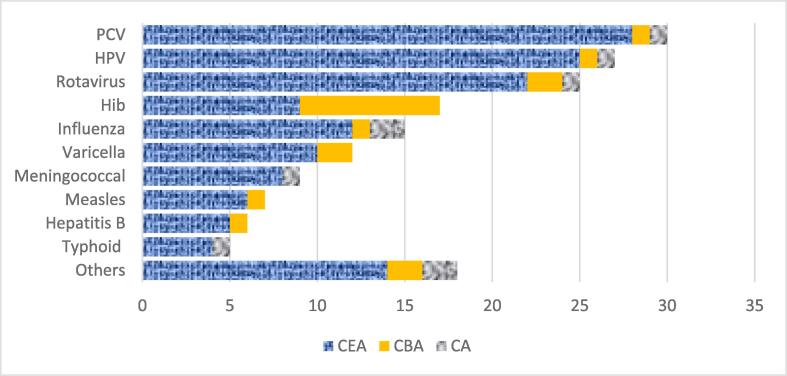
Number of articles with new vaccine cost projections by vaccine and type of study. CA = cost analysis, CBA = cost-benefit analysis, CEA = cost-effectiveness analysis, Hib = haemophilus influenzae type b, HPV = human papillomavirus, and PCV = pneumococcal conjugate vaccine; 'Others' includes the following vaccines: COVID-19, measles-rubella, rubella, oral cholera, pentavalent, polio, and yellow fever. (For interpretation of the references to colour in this figure legend, the reader is referred to the web version of this article.)

Fig. 2 shows the number of articles with new vaccine cost projections by vaccine and study type. The articles were more likely to focus on the following vaccines: pneumococcal conjugate (PCV) (30 studies), HPV (27 studies) and rotavirus (25 studies). Fewer studies were found on newer vaccines such as typhoid and COVID-19. The 171 studies were published between 1990 and 2022.

The articles with new vaccine cost projections were cost-effectiveness analyses (84 %), cost-benefit analyses (11 %) or cost analyses (5 %). When the studies were disaggregated by vaccine, the majority of these were also cost-effectiveness analyses with the exception of Hib vaccine which had equal number of cost-effectiveness and cost-benefit studies.

Most studies were conducted in high- and middle-income countries. Approximately half of the studies were conducted in high-income countries (per capita income US$13,205 or greater [11]) while 39 % were from middle-income countries (18 % in upper middle-income countries with per capita income US$4,256 to US$13,205 [11] and 21 % in lower middle-income countries with per capita income US$1,086 to US$4,255 [11]) (Appendix 3). The remaining 11 % analyzed new vaccine costs in low-income countries (per capita income US$1,085 or less [11]).

Some differences in the type of pathogens studied were also found by country income level. Most studies on influenza vaccine introduction took place in high-income countries while studies on PCV and varicella had a large share of the studies conducted in upper middle-income countries. HPV and Hib vaccine introduction studies were conducted almost evenly in high-, upper middle-, and low-income countries. Studies on rotavirus vaccine introduction, on the other hand, were most common among lower middle- and low-income countries. In low-income countries, many of the studies focused on vaccines preventing diseases commonly occurring in tropical countries such as meningococcal, hepatitis B, and typhoid.

### 3.3 Objectives of new vaccine cost projections

Table 1 summarizes the objectives of the studies with new vaccine cost projections. The most common objective was to estimate the cost-effectiveness of introducing the new vaccine, followed by assessing the impact and economic outcomes.

**Table 1:** Objectives of studies with new vaccine cost projections by vaccine.

Vaccine	Evaluate the cost-effectiveness (# of articles)	Compare costs and benefits (# of articles)	Estimate total cost (# of articles)	Assess impact and economic outcomes (# of articles)	Estimate the impact of changing presentation of vaccine (# of articles)	Other (# of articles)
Pneumococcal	17			12	1	
Human papillomavirus (HPV)	17	1		7	1	1 paper’s main objective is to introduce a costing tool
Rotavirus	10	2	1	11	1	
Haemophilus influenzae type b (Hib)	8	6		2	1	
Influenza	10	1	2	1	1	
Varicella	8	2		2		
Meningococcal	6			1	2	
Measles	4	1		1	1	
Typhoid	3		1	1		
Polio	1	1		2		
Oral cholera	3	1				
Hepatitis B	4	1		1		
COVID-19	1		1	2		
Measles-rubella				1	1	
Rubella	2					
Yellow fever	1					
Pentavalent			1			
Total	95 (56%)	16 (9%)	6 (4%)	44 (26%)	9 (5%)	1 (1%)

### 3.4 Data collection methods

#### 3.4.1 Sources of vaccine delivery cost data

**Fig. 3:**
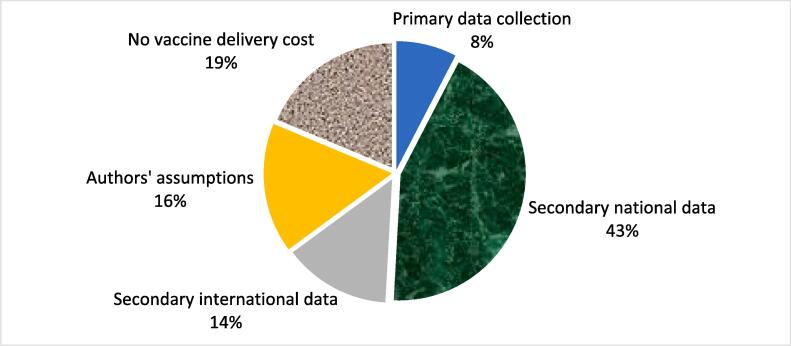
Sources of vaccine delivery cost data in articles.

The most common source of delivery costs in the new vaccine cost projections was secondary national data (43 %) obtained from pilot studies, government fee schedules, and published studies (Fig. 3). Another 16 % used authors’ assumptions on delivery costs and 14 % used cost data from international studies or systematic reviews. In a smaller percentage of articles (8 %), the authors collected the cost data themselves. The remaining 19 % did not include delivery costs in their analysis.

The sources of cost data found in the included studies differed by vaccine (Appendix 4). For PCV, most of the cost data came from two sources: secondary national data (57 %) and authors’ assumptions (27 %). For rotavirus vaccine, the cost data were from four sources: secondary international data (28 %), authors’ assumptions (24 %), secondary national data (24 %) and primary data (16 %). For studies on HPV vaccine introduction, most of the cost data were from secondary country and international data while a smaller number of studies utilized authors’ assumptions and primary data. The cost data sources by vaccine and type of cost analysis are described in detail in Appendix 5.

Over half of the cost-effectiveness and cost-benefit analyses did not estimate detailed vaccine delivery costs (Appendix 6). Instead, they estimated the costs of administering the vaccine only or did not include any delivery costs.

#### 3.4.2 Cost components and sampling methods

Further drilling down into studies that used secondary data in their delivery cost analyses revealed variation in cost components included in studies (Table 2). Around half of the studies (49 %) only included the cost of administering the new vaccine in their calculations – i.e. personnel time of vaccination – and did not include other costs of program support activities such as costs of training, microplanning, cold chain expansion and monitoring and supervision.

**Table 2:** Cost components of vaccine delivery costs in articles that used secondary and primary data by vaccine.

Vaccine	Methods of vaccine delivery costing and sampling	Cost components	Number of articles	Guidance document/costing tool used
**Studies that used secondary cost data**				
Pneumococcal conjugate vaccine (PCV)	Secondary sources	1. Vaccine administration cost only	14	Nil
		2. Incremental system cost *	3	Nil
		3. Secondary ingredients data **	1	Nil
Human papillomavirus (HPV)	Secondary sources	1. Vaccine administration cost only	4	Nil
		2. Incremental system cost *	11	Nil
		3. Secondary ingredients data **	3	Nil
Rotavirus	Secondary sources	1. Vaccine administration cost only	3	Nil
		2. Incremental system cost *	6	Nil
		3. Secondary ingredients data **	4	Nil
Haemophilus influenzae type b (Hib)	Secondary sources	1. Vaccine administration cost only	5	Nil
		2. Incremental system cost *	0	Nil
		3. Secondary ingredients data **	1	Nil
Influenza	Secondary sources	1. Vaccine administration cost only	10	Nil
		2. Incremental system cost *	0	Nil
		3. Secondary ingredients data **	0	Nil
Varicella	Secondary sources	1. Vaccine administration cost only	4	Nil
		2. Incremental system cost *	1	Nil
		3. Secondary ingredients data **	0	Nil
Meningococcal	Secondary sources	1. Vaccine administration cost only	2	Nil
		2. Incremental system cost *	1	Nil
		3. Secondary ingredients data **	1	Nil
Measles	Secondary sources	1. Vaccine administration cost only	2	Nil
		2.Incremental system cost*	1	Nil
		3. Secondary ingredients data **	1	Nil
Typhoid	Secondary sources	1. Vaccine administration cost only	1	Nil
		2. Incremental system cost *	1	Nil
		3. Secondary ingredients data**	2	Nil
Polio	Secondary sources	1. Vaccine administration cost only	2	Nil
		2. Incremental system cost *	1	Nil
		3. Secondary ingredients data**	0	Nil
Oral cholera	Secondary sources	1. Vaccine administration cost only	0	Nil
		2. Incremental system cost *	1	Nil
		3. Secondary ingredients data**	2	Nil
Hepatitis B	Secondary sources	1. Vaccine administration cost only	0	Nil
		2. Incremental system cost *	4	Nil
		3. Secondary Ingredients data**	0	Nil
COVID-19	Secondary sources	1. Vaccine administration cost only	0	Nil
		2. Incremental system cost *	2	Nil
		3. Secondary ingredients data**	0	Nil
Measles-rubella	Secondary sources	1. Vaccine administration cost only	1	Nil
		2. Incremental system cost *	1	Nil
		3. Secondary ingredients data **	0	Nil
Rubella	NA	NA	NA	NA
Yellow fever	Secondary sources	1. Vaccine administration cost only	0	Nil
		2. Incremental system cost *	1	Nil
		3. Secondary ingredients data**	0	Nil
Pentavalent	NA	NA	NA	NA
Total		1. Vaccine administration cost only	**48 (49%)**	
		2. Incremental system cost *	**34 (35%)**	
		3. Secondary ingredients**	**15 (15%)**	
**Studies with primary cost data collection**				
**HPV**				
Hutubessy 2012 [16]	Micro-costing; data collected at national level and health facility visits	Vaccine, injection supplies, personnel (time and salaries), per diems, microplanning, training, social mobilization, cold chain, monitoring and supervision	1	WHO Cervical Cancer Prevention and Control Costing Tool: HPV vaccination module [17]
**Rotavirus**				
Madsen 2014 [18]	Micro-costing; no sampling	Vaccine, cold chain, transportation, personnel (time and salaries), distribution, surveillance, monitoring, training, advocacy, social mobilization, information, education and communication, sensitization, shipping (in % of vaccine cost); and wage data from WHO-CHOICE	4	WHO guidelines for estimating cost of introducing new vaccines into the national immunization system in 2002 [4]
Javanbakht 2015 [19]	Micro-costing; no sampling			WHO guidelines for estimating cost of introducing new vaccines into the national immunization system in 2002 [4]
Ruhago 2015 [20]	Micro-costing and top-down allocations in 2 districts			Modified WHO and UN HIV and AIDS costing tool [20]
Pempa 2020 [21]	Micro-costing; no sampling			UNIVAC [22]
**Hib**				
Ginsberg 1993 [23]	Micro-costing; no sampling	Vaccine, personnel time, health education and training	1	Nil
**Influenza**				
Pecenka 2017 [24]	Data collected from primary and secondary sources at national, zonal/regional, district and facility levels	Introduction (planning, training, social mobilization, information, education and communication), personnel (time and salaries), vaccine, allowances, injection supplies, distribution, supervision and monitoring, and cold chain	2	WHO seasonal influenza immunization costing tool [26]
De Haas 2021 [25]	Micro-costing; no sampling			
**Varicella**				
Esmaeeli 2017 [27]	Data collected from Ministry of Health and Medical Education; no sampling	Vaccines, distribution system, cold chain, surveillance, monitoring, training, and maintenance	1	WHO guidelines for estimating cost of introducing new vaccines into the national immunization system in 2002 [4]
**Measles**				
Babigumira 2011 [28]	Micro-costing; survey of health centers in 4 districts	Vaccine, personnel salaries, transport, social mobilization, training, monitoring, surveillance, and cold chain fuel and maintenance	1	Guidelines for measles eradication study (Levin et al., 2011 [29])
**Typhoid**				
Debellut 2022 [30]	Data collected at central level and 4 districts	Introduction (planning, training and sensitization) and recurrent costs (vaccine procurement, social mobilization, service delivery (personnel time and per diems), supervision and monitoring)	1	WHO costing tool for typhoid vaccine introduction [31], Harvard guidelines on costing in 2020 [32], WHO guidelines for estimating cost of introducing new vaccines into the national immunization system in 2002 [4], and the Common Approach guidelines [33]
**Oral cholera**				
Cookson 1997 [34]	Micro-costing; no sampling	Vaccine, personnel (time and salaries), publicity, materials, transport and cold chain	1	Nil

*Incremental system costs are taken from Comprehensive Multiyear Plans (cMYPs), the Immunization Costing Network (ICAN), Global Immunization Vision and Strategy (GIVS), the Center for Disease Control (USCDC), and costing reviews; **Ingredients include cost components such as training, communication, supervision, cold chain, transportation and supplies; NA = not applicable since no vaccine delivery cost included.

Another third of studies employed a lump sum of incremental costs of an immunization program and included cost components of introducing new vaccines such as introduction (or start-up) costs, costs of cold storage and transportation. These studies either used data from another country (e.g., Llave et al., 2022 [12]), used data from a demonstration project (e.g., Vodicka et al., 2022 [13]), or made an assumption of the costs of vaccine delivery and varied it in the analysis (e.g., Sharma 2012 [14]).

The remaining 15 % of studies had more detailed costing in their analyses and gathered costs of individual cost components. These studies included costs gathered from secondary sources on cost components such as personnel time, introduction activities (training and communication), cold chain equipment and storage, cold chain maintenance, supervision, and surveillance (e.g., Zhang 2021 [15]).

Authors of twelve (8 %) studies with new vaccine cost projections collected primary cost data (Table 2). Four of these studies were about rotavirus vaccine, while the others were studies about influenza, Hib, HPV, measles, oral cholera, typhoid conjugate, and varicella. All twelve studies utilized micro-costing (ingredients costing) to gather primary cost data. Ten studies (83 %) used guidance documents and/or costing tools to guide their costing process.

The cost components included were similar for most new vaccine introduction studies with detailed primary data collection. Most studies estimated some introduction costs such as training (91 %) and social mobilization (73 %) but fewer studies included planning activities (27 %) (Appendix 7). Similarly, most studies included recurrent costs such as vaccine procurement (100 %), personnel time (88 %), and transport (75 %) but fewer studies included other costs such as monitoring and supervision (50 %) and surveillance (33 %). A majority of studies included some capital costs such as the purchase of cold chain equipment (73 %).

### 3.5 Methods of analyses

Table 3 shows the analytical methods employed in the articles with new vaccine cost projections. Most studies (98 %) projected the cost of purchasing vaccines through estimation of the size of target population multiplied by coverage and price per dose. A few studies conducted parity pricing instead so that they could compare two vaccine products that were under consideration by governments for introduction. Other studies used sensitivity analyses to vary the costs of the vaccine since the price per dose had not yet been set by the manufacturers or other source of procurement. Only 12 % of the studies specified that they had included freight, insurance, and handling charges in their calculations.

**Table 3:** Methods of analysis used in new vaccine cost projections.

Vaccine	Vaccine cost (# of articles)	Delivery cost analysis (# of articles)	Perspective (# of articles)	Discount rate (# of articles)	Data uncertainty (# of articles)
Pneumococcal conjugate vaccine (PCV)	VP (27)	Aggregate (24)	Societal (15)	3% (22)	One-way (12)
	VP + FIC (1)	Micro (2)	Health system (8)	3.5% (3)	Probabilistic (17)
	Varied cost (2)	Varied HSC (1)	Government (5)	4% (1)	None (1)
		No delivery cost (3)	Not reported (2)	5% (4)	
Human papillomavirus (HPV)	VP (23)	Aggregate (17)	Societal (9)	3% (17)	One-way (16)
	VP+FIC (4)	Micro (4)	Health system (11)	3.5% (2)	Probabilistic (6)
		No delivery costs (6)	Government (3)	5.0% (4)	None (5)
			Not reported (4)	5.3% (1)	
				Not reported (3)	
Rotavirus	VP (11)	Aggregate (15)	Societal (8)	2% (2)	One-way (14)
	VP + FIC (6)	Micro (7)	Health system (10)	3% (16)	Probabilistic (11)
	Varied cost (7)	Top-down (1)	Government (5)	5% (5)	
	Not included (1)	No delivery cost (2)	Not reported (2)	Not reported (2)	
Haemophilus influenzae type b (Hib)	VP (17)	Aggregate (9)	Societal (8)	2% (1)	One-way (10)
		Micro (2)	Health system (1)	3% (7)	Probabilistic (4)
		No delivery cost (6)	Not reported (8)	5% (6)	None (3)
				6% (1)	
				Not reported (2)	
Influenza	VP (14)	Aggregate (12)	Societal (9)	3% (6)	One-way (9)
	VP+FIC (1)	Micro (2)	Health system (2)	3.5% (2)	Probabilistic (6)
		No delivery costs (1)	Government (2)	5% (1)	
			Not reported (2)	Not reported (6)	
Varicella	VP (11)	Aggregate (7)	Societal (6)	3% (5)	One-way (8)
	VP+FIC (1)	Micro (1)	Health system (3)	3.5% (2)	Probabilistic (2)
		No delivery costs (4)	Government (2)	5% (2)	None (2)
			Not reported (1)	6% (1)	
				Not reported 2	
Meningococcal	VP (8)	Aggregate (6)	Societal (6)	3% (5)	One-way (3)
	VP+FIC (1)	Micro (2)	Health system (3)	4% (1)	Probabilistic (6)
		No delivery costs (1)		5% (2)	
				Not reported (1)	
Measles	VP (5)	Aggregate (4)	Societal (2)	1.5 (1)	One-way (3)
	VP+FIC (2)	Micro (2)	Health system (2)	3% (2)	Probabilistic (3)
		No delivery costs (1)	Government (1)	5% (1)	None (1)
			Not reported (2)	10% (1)	
				Not reported (2)	
Typhoid	VP (3)	Aggregate (3)	Societal (2)	3% (5)	One-way (1)
	VP + FIC (2)	Micro (2)	Health system (2)		Probabilistic (3)
			Government (1)		None (1)
Polio	VP (4)	Aggregate (3)	Societal (4)	3% (2)	One-way (3)
		No delivery cost (1)		Not reported (2)	Probabilistic (1)
Oral cholera	VP (3)	Aggregate (2)	Societal (1)	3% (2)	One-way (2)
	VP + FIC (1)	Micro (2)	Health system (1)	5% (1)	Probabilistic (2)
			Not reported (2)	10% (1)	
Hepatitis B	VP (5)	Aggregate (1)	Societal (1)	3% (4)	One-way (4)
	Not included (1)	No delivery cost (5)	Health system (4)	8% (1)	None (2)
			Not reported (1)	Not reported (1)	
COVID-19	VP (2)	Aggregate (3)	Health system (3)	3% (2)	One-way (3)
	Varied cost (2)	Micro (1)	Not reported (1)	3.5% (1)	Probabilistic (1)
				Not reported (1)	
Measles-rubella	VP (2)	Aggregate (2)	Societal (2)	3% (1)	One-way (1)
				Not reported (1)	Probabilistic (1)
Rubella	VP (2)	No delivery costs (2)	Not reported (2)	Not reported (2)	One-way (1)
					None (1)
Yellow fever	VP + FIC (1)	Micro (1)	Government (1)	3% (1)	Probabilistic (1)
Pentavalent	VP (1)	Aggregate (1)	Government (1)	Not reported (1)	One-way (1)
Total	VP (138, 81%)	Aggregate (109, 64%)	Societal (71, 42%)	3% (97, 57%)	One-way (90, 53%)
	VP+FIC (20, 12%)	Micro (28, 16%)	Health system (52, 30%)	3.5% (10, 6%)	Probabilistic (63, 37%)
	Varied (11, 6%)	Top-down (1, 1%)	Government (21, 12%)	5% (26, 15%)	None (18, 10%)
	Not included (2, 1%)	Varied HSC (1, 1%)	Not reported (27, 16%)	Other (12, 7%)	
		No delivery costs (32, 19%)		Not reported (26, 15%)	

VP = vaccine price, FIC = costs of freight, insurance and charges, HSC = health system costs or program support activity costs.

Two-thirds of the studies used aggregate costing to estimate delivery costs. That is, the studies used an already calculated lump sum of delivery cost (with or without program support activity or health system costs) while another 15 % conducted micro-costing and added together the values of cost components, e.g., training, social mobilization, personnel, supplies, transport, supervision, and monitoring and evaluation. One study used top-down costing where costs were allocated by percentages to input categories.

Most studies (84 %) reported the perspectives that they used in the analyses. The most common perspective was societal (43 %), followed by health system (29 %). In most studies, there was not enough information to identify whether a financial or economic costing approach was used.

### 3.6 Characterization of data uncertainty on cost

The majority of studies included sensitivity analyses to characterize cost data uncertainty in the variable values. Some 53 % were one-way sensitivity analyses, 38 % were probabilistic sensitivity analyses and 9 % did not conduct any analysis of uncertainty.

### 3.7 Use cases of cost estimates

Appendix 8 shows the use cases for the studies with new vaccine cost projections. Slightly over two-thirds of studies (69 %) were conducted to generate evidence on cost-effectiveness or cost-benefit analysis to assist policymakers in decision-making on introduction of a new vaccine. The rest of the studies were conducted to determine which alternative vaccine is more cost-effective (16 %), which vaccine service delivery strategy is more cost-effective or cost-beneficial (11 %), and what the estimated cost would be for introducing the vaccine to assist in planning and advocacy (5 %).

## 4 Discussion

This review showed that most peer-reviewed studies with new vaccine cost projections were cost-effectiveness analyses while a smaller proportion were cost-benefit and cost analyses. A majority of cost-effectiveness and cost-benefit studies only estimated partial vaccine delivery cost in their analyses, i.e., they included vaccine costs with or without administration costs in their estimates and left out other program support activities costs. These studies often focused more on the modeling of cost-effectiveness of the new vaccine and disease treatment costs averted than on accurate estimation of vaccine delivery costs. The five percent of studies that were cost analyses, on the other hand, had the most thorough descriptions of vaccine delivery costs and were more likely to include several cost components rather than aggregate measures. One of the reasons for this might be that cost analyses usually followed guidance documents and/or used costing tools in their analyses.

Among the studies that included vaccine delivery costs in their analyses, the choice of cost components varied widely. Some described cost components for introduction and implementation activities, others included a lump sum for incremental health system costs, and others included only cost estimates of administering the vaccine. The studies that employed primary data collection included many of the same cost components – vaccine, health personnel time and salaries, transport, social mobilization training and cold chain expansion. However, almost half of these did not include costs of monitoring and supervision, which is a key activity required for the introduction of new vaccines. The majority of studies also did not include introductory planning activities. This suggests that having a list of both necessary and highly recommended cost components that should be included in a new vaccine projection would be helpful for researchers to consider in their analyses.

Most of the studies with detailed costing reported following guidance documents and/or using costing tools in their analyses. This finding suggests that making available updated guidance documents and costing tools can aid researchers to include comparable cost components. Other articles did not have clear descriptions of the costing methods, particularly those that used aggregate costing measures of vaccine delivery. Some cited other studies for the costs that they used without explanation, made an assumption about the amount of the vaccine delivery cost, or used a lump sum from a systematic review. This suggests that guidelines should recommend researchers to improve their reporting on costing methods, and use secondary materials if detailed costing is not possible.

More than 90 % of studies took part in high- and middle-income countries while only 9 % focused on low-income countries. One reason for the majority of studies being in high- and middle-income countries may be because new vaccine introductions in high- and middle-income countries are less likely to be subsidized. Their governments are more interested in the affordability and cost-effectiveness of the new vaccines than those in low-income countries that are eligible for funding from organizations such as Gavi. Another reason may be that fewer health economists are from these settings. However, more studies should be encouraged in low-income countries to assist policymakers in evaluating the benefits and affordability of introducing new vaccines and in planning activities ahead of the introduction.

The vaccines that had economic evaluations were more likely to focus on newer antigens that have more expensive presentations, i.e., pneumococcal, HPV, rotavirus and Hib. Few studies have been conducted on COVID-19 vaccines since these have only recently become available for introduction. On the other hand, fewer studies were conducted for older and less costly vaccines such as hepatitis B, yellow fever and polio.

Our study had some limitations. First, we did not conduct the study selection and data extraction in duplicate although the authors did plan to discuss the selection of the articles if there were any uncertainties. Second, we did not include gray literature due to time constraints and concerns about the quality of the articles, so we may have missed some relevant literature.

## 5 Conclusions

In conclusion, with approximately one-third of new vaccine cost projections having detailed or a lump sum of incremental vaccine delivery costs and a minority not including any vaccine delivery costs in their analyses, the review showed that a majority of studies have underestimated the costs of introducing the vaccines in the economic analyses. It also suggests that an updated guidance document on calculation of vaccine delivery costs could assist new vaccine costing studies to be more comparable.

Disclaimer: The opinions expressed in this article are those of the authors and do not necessarily represent the decisions, official policy or opinions of the WHO.

Authors’ contributions

AL and KHTY conceptualized and designed the study, and interpreted the data. AL collected and reviewed the data, conducted the analysis, and drafted the initial manuscript. AL, KHTY and RH contributed to the critical revision of the manuscript, read and approved the final manuscript.

## CRediT authorship contribution statement

**Ann Levin:** Conceptualization, Data curation, Formal analysis, Investigation, Methodology, Validation, Visualization, Writing – original draft, Writing – review & editing. **Karene Hoi Ting Yeung:** Conceptualization, Formal analysis, Investigation, Methodology, Supervision, Writing – review & editing. **Raymond Hutubessy:** Conceptualization, Supervision, Writing – review & editing.

## Declaration of competing interest

The authors declare the following financial interests/personal relationships which may be considered as potential competing interests: Ann Levin reports financial support was provided by the World Health Organization.

## Data Availability

This is a systematic review and all data included was from the electronic database and all references have been included in the appendices.

## Footnotes

1 Although we originally planned to search the databases of Embase and Medline, we were unable to get access to these.
