# Supplementary material for: Systematic review of cost projections of new vaccine introduction
Source: Vaccine. 2024 Feb 15;42(5):1042–50. doi: 10.1016/j.vaccine.2024.01.024 (PMC10911080; doi:10.1016/j.vaccine.2024.01.024)
Supplement: Supplementary data 1 [file mmc1.pdf]

Appendices of “Systematic Review of Cost Projections of New Vaccine Introduction”

Authors: Ann LEVIN, Karene Hoi Ting YEUNG, Raymond HUTUBESSY

DOI: 10.1016/j.vaccine.2024.01.024

Contents

Appendix 1. Full search strategy of the systematic review 2

Appendix 2. Articles included in the systematic review 3

Appendix 3. Figure on the number of articles with new vaccine cost projections by vaccine and income level 13

Appendix 4. Figure on the sources of vaccine delivery cost data in articles by vaccine 13

Appendix 5. Cost data sources by vaccine and type of cost analysis 14

Appendix 6. Figure on the studies by type of economic evaluation and completeness of vaccine delivery cost 16

Appendix 7. Figure on common cost components of vaccine delivery costs included in studies with primary data collection 16

Appendix 8. Use cases for the new vaccine cost projection studies by vaccine 17

Appendix 1. Full search strategy of the systematic review

Databases searched: PubMed, Cochrane Open Access, Mendeley and Google Scholar

Dates of search: 15-16 June 2022

Electronic search strategy for PubMed:

	Search words	Limits (filter, limits, refine)
#1	coronavirus disease 2019 OR COVID-19 OR diphtheria-pertussis-tetanus OR DPT OR haemophilus influenzae type b OR Hib OR hepatitis B OR human papillomavirus OR HPV OR seasonal influenza OR measles-rubella OR meningococcal OR oral cholera OR pneumococcal OR PCV OR polio OR rotavirus OR RTS,S OR typhoid conjugate OR varicella OR yellow fever	
#2	vaccine cost OR cost projection OR costing OR vaccine OR vaccination OR delivery costs OR immunization	
#3	#1 AND #2	
#4	#3	Publication dates: 1990 – 15 June 2022<br>English language

Appendix 2. Articles included in the systematic review

Author	Title	Type of Cost Analysis	Income Level	Source of vaccine delivery cost
**Pneumococcal**				
Ansaldi et al. 2020 Pathogens	Estimating the clinical and economic impact of switching from the 13-valent pneumococcal conjugate to the 10-valent pneumococcal conjugate vaccine in Italy	CEA	HI	Assumptions
Ayieko et al. 2013 PLoS One	Assessment of Health Benefits and Cost-Effectiveness of 10-valent and 13-valent pneumococcal conjugate vaccination in Kenyan children	CEA	LMI	Secondary national data
Bakir et al. 2012 BMC Health Services Research	Cost-effectiveness of new pneumococcal conjugates vaccines in Turkey: a decision analytical model	CEA	UMI	Assumptions
Bergman et al. 2008 Scand J Infect Dis	Cost-effectiveness analysis of a universal vaccination programme with the 7-valent pneumococcal conjugate vaccine in Sweden	CEA	HI	Assumptions
Che et al. 2014 BMC Health Serv Res	Modeling the impact of the 7-valent pneumococcal conjugate vaccine in Chinese infants	CEA	UMI	Secondary national data
Constenla et al. 2008 Rev Panam Salud Publica	Economic impact of pneumococcal conjugate vaccination in Brazil, Chile, and Uruguay	CEA	UMI/HI	Secondary national data
Ess et al. 2003 Vaccine	Cost-effectiveness of a pneumococcal conjugate immunization program for infants in Switzerland	CEA	HI	Secondary national data
Farinas et al. 2021 International Journal of Infectious Diseases	Cost-effectiveness of introducing a domestic pneumococcal conjugate vaccine into the Cuban national immunization programme	CEA	UMI	Secondary national data
Giglio et al. 2010 Vaccine	Cost effectiveness of the CRM-based 7-valent pneumococcal conjugated vaccine in Argentina	CEA	UMI	International data
Krishnamorthy et al. 2019 Vaccine	Impact and cost-effectiveness of pneumococcal conjugate vaccine in India	CEA	LMI	Secondary national data
Lebel et al. 2003 Clin Infect Dis	A pharmacoeconomic evaluation of 7-valent pneumococcal conjugate vaccine in Canada	CEA	HI	Secondary national data
Lee et al. 2009 Value Health	Economic Evaluation of Universal Infant Vaccination with 7vPCV in Hong Kong	CEA	HI	Secondary national data
Lieu et al. 2000 JAMA	Projected Cost-effectiveness of pneumococcal conjugate vaccination of health infants and young children	CEA	HI	Secondary national data
Mo et al. 2016 Pediatric Infectious Diseases J	Cost-effectiveness and Health benefits of pediatric 23-valent pneumococcal polysaccharide vaccine, 7 valent pneumococcal conjugate vaccine and forecasting 13-valent pneumococcal conjugate vaccine in China	CEA	UMI	Secondary national data
Pecenka et al 2021 BMJ Global Health	Pneumococcal conjugate vaccination in the Gambia	CEA	LI	Primary data collection
Stoecker et al. 2016 J Gen Intern Med	Incremental Cost-Effective of 13-valent Pneumococcal conjugate Vaccine for Adults Age 50 Years and Older in the US	CEA	HI	Secondary national data

Author	Title	Type of Cost Analysis	Income Level	Source of vaccine delivery cost
Pugh et al. 2019 Infectious Disease Ther	Cost-effectiveness of the pneumococcal conjugate vaccine versus no vaccination for a national immunization program in Tunisia or Algeria	CEA	LMI	Assumptions
Pugh et al. 2020 Infect Dis Ther	Estimating the Impact of Switching from a Lower to Higher Valent Pneumococcal Conjugate Vaccine in Colombia, Finland, and the Netherlands: A Cost-Effectiveness Analysis	CEA	UMI/HI	Secondary national data
Shafie et al. 2020 Human Vaccines & Immunotherapeutics	Estimating the population health and economic impacts of introducing a pneumococcal conjugate vaccine in Malaysia – an economic evaluation	CEA	UMI	No delivery costs
Shiragami et al. 2014 Infect Dis Ther	Cost-effectiveness evaluation of the 10-valent Pneumococcal Non-typeable Hib Protein D Conjugate Vaccine and 13 valent Pneumococcal vaccine in Japanese Children	CEA	HI	Secondary national data
Talbird et al. 2010 Vaccine	Outcomes and costs associated with PHiD-CV, a new protein D conjugate pneumococcal vaccine, in four countries	CBA	UMI/HI	No delivery costs
Thorrington et a. 2018 PLoS One	Impact and cost-effectiveness of different vaccination strategies to reduce the burden of pneumococcal disease among elderly in the Netherlands	CEA	HI	Assumptions
Van Hoek et al. 2012 Vaccine	The cost-effectiveness of a 13-valent pneumococcal conjugate vaccination for infants in England	CEA	HI	Secondary national data
Van Hoek et. al. 2016 PLoS One	Cost-effectiveness of vaccinating immunocompetent >= 65 years old with the 13-valent pneumococcal conjugate vaccine in England	CEA	HI	Secondary national data
Wang et al. 2017 Cost Eff Resour Alloc	Cost-effectiveness analysis of a universal mass vaccination program with a PHiD-CV 2+1 schedule in Malaysia	CEA	UMI	No delivery costs
Weycker et al. 2012 Vaccine	Public health and economic impact of 13-valent pneumococcal conjugate vaccine in US adults aged >= 50 years	Cost analysis	HI	Secondary national data
Wu et al. 2012 Value in Health	Cost-effectiveness analysis of pneumococcal conjugate vaccine in Taiwan: A transmission dynamic modeling approach	CEA	HI	Assumptions
Wu et al. 2016 Human Vaccines & Immunotherapeutics	Cost-effectiveness analysis of infant universal routine pneumococcal vaccination in Malaysia and Hong Kong	CEA	UMI/HI	Assumptions
Zhang et al. 2014 Value Health Reg Issues	Cost-effectiveness Analysis of pneumococcal vaccination with the pneumococcal polysaccharide NTHi Protein D Conjugate Vaccine in the Philippines	CEA	LMI	No delivery costs
Zhang et al. 2018 Human Vaccines & Immunotherapeutics	Cost-effectiveness analysis of infant pneumococcal vaccination with PHID-CV in Korea	CEA	HI	Assumptions
**Human Papillomavirus**				

Author	Title	Type of Cost Analysis	Income Level	Source of vaccine delivery cost
Aljunid et al. 2010<br>Asian Pac J Cancer Prev	Burden of disease associated with cervical cancer in Malaysia and potential costs and consequences of HPV vaccination	CEA	UMI	No delivery costs
Anwari et al. 2020<br>Vaccine	Potential health impact and cost-effectiveness of bivalent human papillomavirus vaccination in Afghanistan	CEA	LI	International data
Bahr et al. 2019<br>East Mediterr Health J	Cost-benefit analysis of a projected national human papilloma virus vaccination programme in Lebanon	CBA	LMI	No delivery costs
Boiron et al. 2016<br>BMC Infect Dis	Estimating the cost-effectiveness profile of a universal vaccination programme with a nine-valent HPV vaccine in Austria	CEA	HI	Secondary national data
Chesson et al. 2011<br>Vaccine	The cost-effectiveness of male HPV vaccination in the United States	CEA	HI	Secondary national data
Cheung et al. 2021<br>Cost Eff Resour Alloc	The impact and cost-effectiveness of 9-valent human papillomavirus vaccine in adolescent females in Hong Kong	CEA	HI	Secondary national data
Cody et al. 2021<br>BMC Infect Dis	Public health impact and cost-effectiveness of routine and catch-up vaccination of girls and women with a nine-valent HPV vaccine in Japan: a model-based study	CEA	HI	Secondary national data
Dasbach et al. 2010<br>J Med Econ	The cost effectiveness of a quadrivalent human papillomavirus vaccine in Hungary	CEA	HI	No delivery costs
Datta et al. 2019<br>BMC Infect Disease	Assessing the cost-effectiveness of HPV vaccination strategies for adolescent girls and boys in the UK	CEA	HI	Secondary national data
Diaz et al. 2008<br>Br J Cancer	Health and economic impact of HPV 16 and 18 vaccination and cervical cancer screening in India	CEA	LMI	International data
Gomez et al. 2014<br>BMC Public Health	Human economic analysis of human papillomavirus vaccines in women of Chile	CEA	HI	Assumptions
Hutubessy et al. 2012<br>BMC Medicine	A case study using the United Republic of Tanzania: costing nationwide HPV vaccine delivery using the WHO Cervical Cancer Prevention and Control Costing Tool	CA	LMI	Primary data collection
Isshiki et al. 2014<br>Asian Pac J of Cancer Prevention	HPV vaccination for cervical cancer prevention is not Cost-effective in Japan	CEA	HI	No delivery costs
Kawai et al. 2012<br>BMC Infect Dis	Estimated health and economic impact of quadrivalent HPV vaccination in Brazil using a transmission dynamic model	CEA	UMI	No delivery costs
Kim et al 2007<br>Brit J of Cancer	The value of including boys in an HPV vaccination programme: a cost-effectiveness analysis in a low-resource setting	CEA	UMI	International data
Kim et al. 2021<br>PLoS Med	Human papillomavirus vaccination for adults aged 30 to 45 years in the United States: A cost-effectiveness analysis	CEA	HI	No delivery costs
Lin et al. 2017<br>Clin Infect Dis	Impact and Cost-effectiveness of selective human papillomavirus vaccination of men who have sex with men	CEA	HI	Secondary national data
LLave et al. 2022<br>Vaccine	The cost-effectiveness of human papillomavirus vaccination in the Philippines	CEA	LMI	International data

Author	Title	Type of Cost Analysis	Income Level	Source of vaccine delivery cost
Ma et al. 2021 Human Vaccines and Immunotherapeutics	Modeling the epidemiological impact and cost-effectiveness of a combined schoolgirl HPV vaccination and cervical cancer screening program among Chinese women	CEA	UMI	International data
Mahumud et al 2020 Vaccine	Cost-effectiveness of the introduction of two dose bivalent and quadrivalent HPV vaccination for adolescent girls in Bangladesh	CEA	LMI	International data
Simoens et al. 2021 Front Pharmcol.	Health Impact and Cost-effectiveness of implementing gender-neutral vaccination with the 9-valent human papillomavirus vaccine in Belgium	CEA	HI	No delivery costs
Sharma et al. 2012 BJOG	Cost-effectiveness of human papillomavirus vaccination and cervical cancer screening in Thailand	CEA	UMI	Assumptions
Uuskula et al. 2013 BMC Infect Dis	The epidemiological and economic impact of a quadrivalent human papillomavirus vaccine in Estonia	CEA	HI	Secondary national data
Vodicka et al. 2022 Vaccine	The projected cost-effectiveness and budget impact of HPV vaccine introduction in Ghana	CEA	LMI	Secondary national data
Vorno et al. 2017 Vaccine	Cost-effectiveness of HPV vaccination in the context of high cervical cancer incidence and low screening coverage	CEA	HI	Assumptions
Walwyn et al. 2015 Vaccine	Cost-effectiveness of HPV vaccination in Belize	CEA	UMI	Secondary national data
Wolff et al. 2018 Vaccine	Cost-effectiveness of sex-neutral HPV vaccination in Sweden, accounting for herd immunity and sexual behaviour	CEA	HI	Secondary national data
**Rotavirus**				
Alwaidy et al. 2014 BMC Infect dis	Cost-effectiveness of a pentavalent rotavirus vaccine in Oman	CEA	HI	Assumptions
Anderson et al. 2020 PLoS One	Effects of geographic and economic heterogeneity on the burden of rotavirus diarrhea and the impact and cost-effectiveness of vaccination in Nigeria	CEA	LMI	Secondary national data
Bakir et al. 2013 Vaccine	Estimating and comparing the clinical and economic impact of paediatric rotavirus vaccination in Turkey using a simple vs an advanced model	CEA	UMI	No delivery costs
Constenla et al. 2009 Rev Panam salud Publica	Economic impact of a rotavirus vaccination program in Mexico	CBA	UMI	Assumptions
Cui et al. 2016 BMC Infect Dis	Cost -effectiveness analysis of rotavirus vaccination in China: Projected possibility of scale-up from the current domestic option	CEA	UMI	No delivery costs
Diop et al. 2015 Vaccine	Estimated impact and cost-effectiveness of rotavirus vaccination in Senegal	CEA	LMI	Secondary national data
Esposito et al. 2011 Clin Infect Dis	Projected impact and cost-effectiveness of a rotavirus vaccination program in India, 2008	CEA	LMI	International data
Fisman et al. 2012 Vaccine	Effectiveness and cost-effectiveness of pediatric rotavirus vaccination in British Columbia: a model-based evaluation	CEA	HI	Secondary national data

Author	Title	Type of Cost Analysis	Income Level	Source of vaccine delivery cost
Itzler et al. 2013<br>J Med Econom	Cost-effectiveness of a pentavalent rotavirus vaccine in Japan	CEA	HI	Secondary national data
Javanbakht et al. 2015<br>Vaccine	Cost-effectiveness analysis of the introduction of rotavirus vaccine in Iran	CEA	LMI	Primary data collection
Koksal et al 2017<br>J Microbiol Immunol Infect	Cost-effectiveness of rotavirus vaccination in Turkey	CEA	UMI	Assumptions
Kurosawa et al. 2021<br>Pediatric Infectious Dis	Cost-utility of Rotavirus vaccines including the latest evidence and data as of June 2020 in Japan	CEA	HI	Assumptions
Liu et al. 2012<br>Vaccine	Projected health impact and cost-effectiveness of rotavirus vaccination among children < 5 years of age in China	CEA	UMI	Secondary national data
Lusvan et al. 2019<br>Vaccine	Projected impact, cost-effectiveness, and budget implications of rotavirus vaccination in Mongolia	CEA	LMI	International data
Madsen et al. 2014<br>Tropical Medicine and International Health	Estimating the costs of implementing the rotavirus vaccine in the national immunisation programme: The case of Malawi	CA	LI	Primary data collection
Nantasit et al. 2021<br>Vaccine	Cost-effectiveness and budget impact analyses for the prioritization of the four available rotavirus vaccines in the national programme in Thailand	CEA	UMI	International data
Okafor et al. 2021<br>The Lancet Global Health	Introducing rotavirus vaccine in eight sub-saharan African countries: a cost-benefit analysis	CBA	LI/LMI	International data
Pempa 2020<br>Vaccine	Economic evaluation of rotavirus vaccination in children of Bhutan	CEA	LMI	Primary data collection
Perez-Rubio et al. 2011<br>Le Infezioni in Medicina	Socio-economic modelling of rotavirus vaccination of rotavirus vaccination in Castilla y Leon, Spain	CEA	HI	Secondary national data
Ruhago et al. 2015<br>Cost Effectiveness and Res Allocation	Cost-effectiveness of live oral attenuated human rotavirus vaccine in Tanzania	CEA	LMI	Primary data collection
Tate et al. 2009<br>J Infect Dis	Rotavirus disease burden and impact and cost-effectiveness of a rotavirus vaccination program in Kenya	CEA	LMI	International data
Tate et al. 2011<br>Vaccine	Projected health benefits and costs of pneumococcal and rotavirus vaccination in Uganda	CEA	LI	Assumptions
Villanueva-Uy et al. 2021<br>Vaccine	Cost-effectiveness of rotavirus vaccination in the Philippines	CEA	LMI	International data
Wilopo et al. 2009<br>Vaccine	Economic evaluation of a routine rotavirus vaccination programme in Indonesia	CEA	LMI	Assumptions
Yeung et al. 2021<br>Vaccine	Economic evaluation of the introduction of rotavirus vaccine in Hong Kong	CEA	HI	Secondary national data
**Hib**				
Broughton et al 2007<br>J Public Health	Economic evaluation of Haemophilus influenzae type b vaccination in Indonesia: a cost-effectiveness analysis	CEA	LMI	No delivery costs
Fendrick et al. 1999	Clinical and economic impact of a combination Haemophilus influenzae and	CEA	HI	Secondary national data

Author	Title	Type of Cost Analysis	Income Level	Source of vaccine delivery cost
Arch Pediatr Adolesc Med	Hepatitis B; estimating cost-effectiveness using decision analysis			
Gargano et al. 2015 Prehosp Disaster Med	Pneumonia Prevention during a Humanitarian Emergency: Cost-effectiveness of Haemophilus influenzae Type B Conjugate Vaccine and Pneumococcal Conjugate Vaccine in Somalia	CEA	LI	Secondary national data
Garpenholt et al. 1998 ScandJ Infect Dis.	Economic Evaluation of General Childhood vaccination against Haemophilus influenzae type b in Sweden	CBA	HI	Assumptions
Ginsberg et al 1994 J Epid Community Health	Cost benefit analysis of Haemophilus influenzae type b vaccination programme in Israel	CBA	HI	Primary data collection
Gupta et al. 2012 Health Policy Plan	Cost Effectiveness of Hib type b vaccine introduction in the universal immunization schedule in Haryana State, India	CEA	LMI	No delivery costs
Hussey et al. 1995 S Afr Med J	The costs and benefits of a vaccination programme for Haemophilus influenzae type B disease	CBA	UMI	Assumptions
Jimenez et al. 1999 Pharmaeconomics	Cost Benefit Analysis of Haemophilus influenzae type b vaccination in children in Spain	CBA	HI	Secondary national data
Levine et al. 1993 Am J Epidemiol	Cost Benefit Analysis for the use of Haemophilus influenzae type b in Santiago, Chile	CBA	HI	No delivery costs
Limcangco et al. 2001 Pharmaeconomics	Cost Benefit Analysis of a Haemophilus influenzae type b prevention programme in the Philippines	CBA	LMI	No delivery costs
McIntyre et al. 1994 Aust J Public Health	An economic analysis of alternatives for childhood immunization against Haemophilus influenzae type B disease	CEA	HI	Secondary national data
Moradi-Lakeh M 2021 Int J Prev med	Immunization against Haemophilus influenzae type b in Iran; Cost utility and cost-benefit Analyses	CEA CBA	LMI	No delivery costs
Platonov et al. 2006 Vaccine	Economic evaluation of Haemophilus influenzae type b vaccination in Moscow, Russian Federation	CEA	UMI	No delivery costs
Pokorn et al 2001 Vaccine	Economic Evaluation of Haemophilus influenzae type b vaccination in Slovenia	CBA, CEA	HI	Assumptions
Shin et al. 2008 J Korean Med Sci	Cost Benefit of Haemophilus Influenzae Type B immunization in Korea	CBA	HI	Secondary national data
Trollfors et al. 1994 Scand J Infect Dis	Cost-benefit analysis of general vaccination against Haemophilus influenzae type b in Sweden	CBA	HI	No delivery costs
Zhang et al 2021 BMC Medicine	National and provincial impact and Cost Effectiveness of Haemophilus influenzae type b conjugate vaccine in China: a modeling analysis	CEA	UMI	Secondary national data
**Influenza**				
Beigi 2009 Clinical Infectious Diseases	Economic value of seasonal and pandemic influenza vaccination during pregnancy in the US	CEA	HI	No delivery costs

Author	Title	Type of Cost Analysis	Income Level	Source of vaccine delivery cost
Brogan et al. 2017<br>Human Vaccines and Immunotherapeutics	Cost effectiveness of seasonal quadrivalent vs trivalent influenza vaccination in the US: A dynamic transmission modeling approach	CEA	HI	Secondary national data
Clements 2014<br>Human Vaccin Immunotherapeutics	Cost Effectiveness of universal influenza vaccination with quadrivalent inactivated vaccine in the US	CEA	HI	Secondary national data
de Haas 2021<br>Vaccine	Programme costs for introducing age/gestation-based universal influenza vaccine schedules for young children and pregnant women in Hong Kong	CA	HI	Primary data collection
Duncan et al. 2012<br>Cost Effectiveness and Resource Allocation	Planning influenza vaccination programs in US: a cost benefit model	CBA	HI	Secondary national data
Fisman et al. 2011<br>PLoS One	Estimation of the Health Impact and Cost-effectiveness of influenza vaccination with enhanced effectiveness in Canada	CEA	HI	Secondary national data
Kohli et al 2021<br>Human Vaccine and Immunotherapeutics	The Cost effectiveness of an adjuvanted quadrivalent influenza vaccine in the United Kingdom	CEA	HI	Assumptions
Marchetti et al. 2007<br>Hum Vaccin	Cost effectiveness of adjuvanted influenza vaccination of healthy children 6 to 60 months of age	CEA	HI	Secondary national data
Pecenka 2017<br>PLoS One	Maternal influenza immunization in Malawi: Piloting a maternal influenza immunization program costing tool by examining a prospective program	CA	LI	Primary data collection
Prosser et al. 2006<br>Emerging Infectious	Health benefits, risks, and Cost Effectiveness of influenza vaccination of children in US	CEA	HI	Secondary national data
Salo et al 2006<br>Vaccine	Cost Effectiveness of influenza vaccination of healthy children	CEA	HI	Assumptions
Thorrington et al. 2017<br>BMC Medicine	Cost Effectiveness analysis of quadrivalent seasonal influenza vaccines in England	CEA	HI	Secondary national data
Xu et al. 2014<br>Vaccine	Cost Effectiveness of seasonal inactivated influenza vaccination among pregnant women	CEA	HI	Secondary national data
You et al. 2015<br>Human Vaccines and Immunotherapeutics	Cost Effectiveness of quadrivalent influenza vaccine in Hong Kong – A decision analysis	CEA	HI	Secondary national data
Turner et al. 2006<br>Vaccine	The Cost Effectiveness of influenza vaccination of healthy adults 50-64 years	CEA	HI	Secondary national data
**Varicella**				
Brisson et al. 2022<br>Vaccine	The cost-effectiveness of varicella vaccination in Canada	CEA	HI	No delivery costs
Brisson et al. 2003<br>Archives of disease in Childhood	Varicella vaccination in England and Wales: cost-utility analysis	`CEA	HI	No delivery costs
Burnham et al 1998<br>Mil Med	A Cost Benefit analysis of a routine varicella vaccination program for United States Air Force Academy cadets	CBA	HI	Secondary national data
Domingo et al 1999<br>Vaccine	A cost benefit analysis of routine varicella vaccination in Spain	CBA	HI	No delivery costs

Author	Title	Type of Cost Analysis	Income Level	Source of vaccine delivery cost
Esmaeeli et al 2017<br>Int J of Prev Medicine	Cost Effectiveness of varicella vaccination program in Iran	CEA	LMI	Primary data collection
Lieu et al 1995<br>Pediatrics	Cost effectiveness of varicella serotesting vs presumptive vaccination of school-age children and adolescents	CEA	HI	Secondary national data
Marijam 2022<br>Human Vaccine and Immunotherapeutics	Cost Effectiveness and budget impact of universal varicella vaccination in Russia	CEA	UMI	Secondary national data
Pawaskar et al. 2021<br>PLoS One	Clinical and economic impact of universal varicella vaccination in Norway	CEA	HI	Assumptions
Valentim et al. 2008<br>Vaccine	Cost effectiveness analysis of universal children vaccination against varicella in Brazil	CEA	UMI	International data
Van Hoek et al. 2012<br>Vaccine	The Cost Effectiveness of varicella and combined varicella and herpes zoster vaccination programmes in the United Kingdom	CEA	HI	No delivery costs
Wolfson et al 2019<br>PLoS One	Cost effectiveness analysis of universal varicella vaccination in Turkey using a dynamic transmission model	CEA	UMI	Assumptions
Zhou et al. 2008<br>J Infect Dis	An economic analysis of the universal varicella vaccination program in the US	CEA	HI	Secondary national data
**Meningococcal**				
Arifin et al. 2019<br>Medical Decision Making	Cost Effectiveness of the alternative uses of polyvalent meningococcal vaccines in Niger: an agent-based transmission modeling study	CEA	LI	Assumptions
Bos et al. 2001<br>Vaccine	Health economics of a hexavalent meningococcal outer-membrane vesicle vaccine in children	CEA	HI	Assumptions
Columbini et al. 2015<br>Clinical Infectious Dis	Costs of Neisseria meningitidis Group A Disease and Economic Impact of Vaccination in Burkina Faso	CA	LI	Secondary national data
Delea et al 2017<br>PLoS One	Cost Effectiveness of alternate strategies for childhood immunization against meningococcal disease with monovalent and quadrivalent conjugate vaccines in Canada	CEA	HI	Secondary national data
De Soarez 2011<br>Value in Health	Cost Effectiveness analysis of a universal infant immunization program with meningococcal C conjugate vaccine in Brazil	CEA	UMI	Assumptions
Ginsberg et al. 2016<br>Int J of Public Health	Cost-utility analysis of a nationwide vaccination programme against serogroup B meningococcal disease in Israel	CEA	HI	No delivery costs
Shepard et al. 2005<br>Pediatrics	Cost Effectiveness of conjugate meningococcal vaccination strategies in the US	CEA	HI	Assumptions
Tu et al. 2014<br>Vaccine	Economic evaluation of meningococcal serogroup B vaccination in Ontario	CEA	HI	Secondary national data
**Measles**				

Author	Title	Type of Cost Analysis	Income Level	Source of vaccine delivery cost
Babigumira et al. 2011<br>J Infect Dis	Assessing the cost-effectiveness of measles elimination in Uganda: local impact of a global eradication program	CEA	LI	Primary data collection
Driessen et al 2015<br>Soc Sci and Med	Comparing the health and social protection effects of measles vaccination in Ethiopia	CEA	LI	International data
Ginsberg et al 1990<br>Measles and Poliomyelitis	Cost-benefit analysis of a second dose measles inoculation of children	CBA	HI	No delivery costs
Janusz 2020<br>Vaccine	Measles vaccination of young infants in China	CEA	UMI	Secondary national data
Levin 2007<br>Vaccine	An economic evaluation of thermostable vaccines in Cambodia, Ghana and Bangladesh	CEA	LMI	Secondary national data
Ramsay 2019<br>Eurosurveillance	Cost Effectiveness of measles control during elimination in Ontario	CEA	HI	Secondary national data
Shiel et al 1998<br>Australian and New Zealand J of Public Health	Cost Effectiveness of measles control during elimination in Ontario	CEA	HI	International data
**Typhoid**				
Carias et al. 2015<br>Vaccine	Economic eval. of typhoid vaccination in outbreak setting: Uganda	CEA	LI	Secondary national data
Chauhan et al. 2021<br>Vaccine	Cost Effectiveness of typhoid vaccination in India	CEA	LMI	Secondary national data
Cook et al. 2008<br>Vaccine	The Cost Effectiveness of typhoid Vi vaccination programs: Calculations for four urban sites in four Asian countries	CEA	LMI	International data
Debullut et al. 2022<br>Vaccine	Projecting the cost of introducing typhoid conjugate vaccine in the national immunization Program in Malawi	CA	LI	Primary data collection
Ryckman et al. 2021<br>J Infect Dis	Comparison of Strategies for Typhoid Conjugate Vaccine Introduction in India: A Cost Effectiveness Modeling Study	CEA	LMI	Secondary national data
**Oral cholera**				
Cookson 1997<br>Int. J Epid	A Cost Benefit Analysis of programmatic use of oral cholera vaccine	CBA	UMI	Primary data collection
Khan et al 2018<br>PLoS Neglected Trop Dis	The impact and cost effectiveness of controlling cholera through oral cholera vaccination in Bangladesh	CEA	LMI	Secondary national data
Murray 1998<br>Bull WHO	Cost Effectiveness of oral cholera vaccine in a stable refugee population at risk for epidemic cholera and in a population with endemic cholera	CEA	LI	International data
Zeng et al. 2021<br>Vaccine	Optimizing immunization schedules in endemic cholera regions: cost-effectiveness assessment of vaccination strategies for cholera control in Bangladesh	CEA	LMI	International data
**Polio**				
Griffiths et al 2006<br>Vaccine	The CEA of alternative polio immunization policies in South Africa	CEA	UMI	No delivery costs
Miller et al. 1996<br>JAMA	CEA of incorporating inactivated polio vaccine into routine immunization in the USA	CEA	HI	Secondary national data

Author	Title	Type of Cost Analysis	Income Level	Source of vaccine delivery cost
Tebben et al. 2017<br>MDM Policy Pract	Costs and benefits of including IPV in Outbreak Response in Nigeria	CBA	LMI	International data
Tucker et al. 2001<br>Aust NZ J Public Health	CEA of changing from live OPV to IPV in Australia	CEA	HI	Secondary national data
**Covid**				
Kohli et al 2021<br>Vaccine	Potential public health and economic value of a hypothetical covid-19 vaccine in the US	CEA	HI	Secondary national data
Marco-Franco et al 2021<br>Mathematics	Simplified mathematical modeling of uncertainty: Cost Effectiveness Analysis in Spain	CEA	HI	Secondary national data
Pearson et al. 2021<br>PLoS Med	Covid 19 vaccination in Sindh Province, Pakistan	CEA	LMI	Secondary national data
**Hepatitis B**				
Demicheli et al. 1992<br>J Public Health Med	Cost-benefit analysis of the introduction of mass vaccination against hepatitis B in Italy	CBA	HI	No delivery costs
Gosset et al. 2021<br>Vaccine	Cost-effectiveness of adding a birth dose of Hepatitis B vaccine in the Dafra district of Burkina Faso	CEA	LI	International data
Kim et al. 2006<br>Am J Prev Med	Cost effectiveness of hepatitis B vaccination at HIV counseling and testing sites	CEA	HI	Secondary national data
Klingler et al. 2012<br>Vaccine	CEA of an additional birth dose of Hepatitis B vaccine to prevent perinatal transmission in a medical setting in Mozambique	CEA	LI	International data
Memirie et al 2020<br>Cost Eff Resour Alloc	Introduction of birth dose of hepatitis B virus vaccine to the immunization program in Ethiopia: an economic evaluation	CEA	LI	International data
Szucs et al. 2000<br>Vaccine	Cost- effectiveness of hepatitis A and B vaccination programme in Germany	CEA	HI	Secondary national data
**Measles Rubella**				
Bangs et al. 2022<br>J Pediatric Infect Dis Soc.	The Clinical and Economic Impact of Measles-Mumps-Rubella Vaccinations to prevent Measles Importations from US pediatric Travelers returning from abroad	CEA	HI	Secondary national data
Hyle et al 2019<br>Clin Infect Dis	The Clinical Impact and Cost-effectiveness of Measles-Mumps-Rubella Vaccination to Prevent Measles Importations among International Travelers from the US	CEA	HI	Secondary national data
**Rubella**				
Itatani et al. 2021<br>Vaccines	Model comparisons of the cost effectiveness of rubella vaccination method in Japanese adults	CEA	HI	No delivery costs
Saito et al. 2018<br>IJERPH	Assessing the effectiveness and cost-benefit of test-and vaccinate policy for supplementary vaccination against rubella	CEA, CBA	HI	No delivery costs
**Pentavalent**				
Teimouri et al. 2017<br>Daru	Budget impact analysis of Hib as part of pentavalent vaccine in Iran	CA	LMI	Secondary national data
**Yellow Fever**				
Levin 2007<br>Vaccine	An economic evaluation of thermostable vaccines	CEA	LMI	Secondary national data

CA = cost analysis, CBA = cost-benefit analysis, CEA = cost-effectiveness analysis, HI = high-income, LI = lower-income, LMI = Lower-middle-income, UMI = upper-middle-income

Appendix 3. Figure on the number of articles with new vaccine cost projections by vaccine and income level

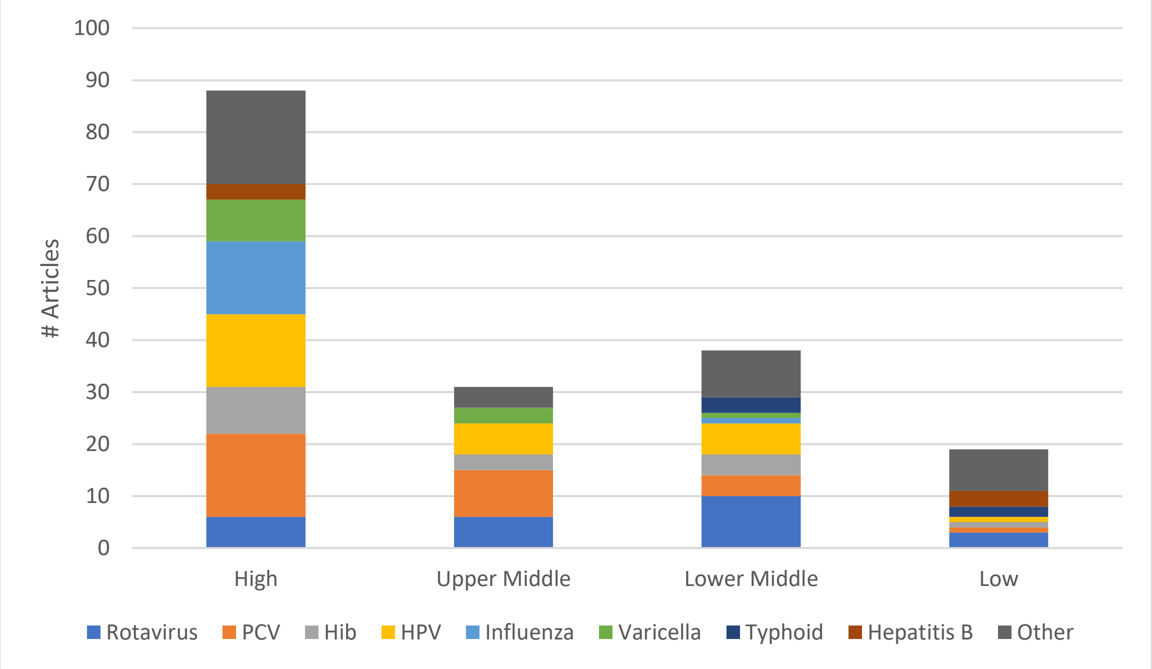

Hib = haemophilus influenzae type b, HPV = human papillomavirus, and PCV = pneumococcal conjugate vaccine; 'Others' includes the following vaccines: COVID-19, measles, measles-rubella, meningococcal, rubella, oral cholera, pentavalent, polio, typhoid, and yellow fever.

Appendix 4. Figure on the sources of vaccine delivery cost data in articles by vaccine

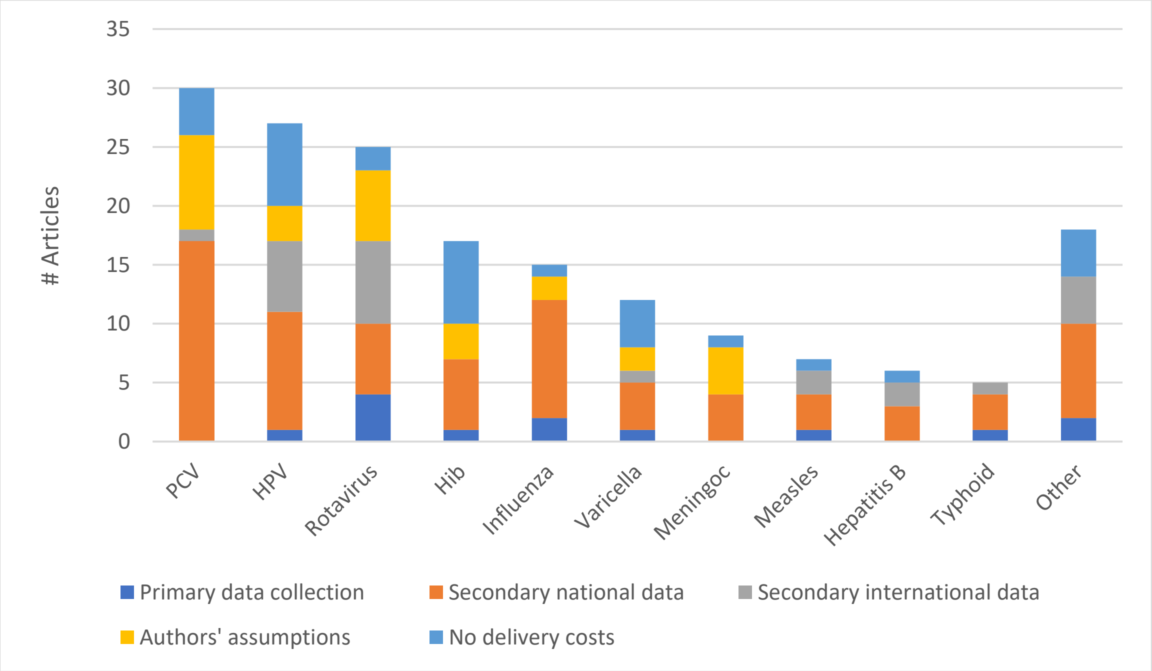

Hib = haemophilus influenzae type b, HPV = human papillomavirus, and PCV = pneumococcal conjugate vaccine; 'Others' includes the following vaccines: COVID-19, measles-rubella, rubella, oral cholera, pentavalent, polio, and yellow fever.

Appendix 5. Cost data sources by vaccine and type of cost analysis

Vaccine	Type of cost analysis	Cost data source (number of studies)
Pneumococcal conjugate vaccine (PCV)	CEA	Secondary national data from pilots, government and publications (16)<br>Secondary international data (1)<br>Assumptions by authors (8)<br>No delivery cost estimated (3)
	CBA	No delivery cost estimated (1)
	CA	Secondary national data from pilots, government and publications (1)
Human papillomavirus (HPV)	CEA	Secondary national data from pilots, government and publications (10)<br>Secondary international data (6)<br>Assumptions by authors (3)<br>No delivery cost estimated (6)
	CBA	No delivery cost estimated (1)
	CA	Primary data collected (1)
Rotavirus	CEA	Primary data collected (3)<br>Secondary national data from pilots, government and publications (6)<br>Secondary international data (6)<br>Assumptions by authors (5)<br>No delivery cost estimated (2)
	CBA	Secondary international data (1)<br>Assumptions by authors (1)
	CA	Primary data collected (1)
Haemophilus influenzae type b (Hib)	CEA	Secondary national data from pilots, government and publications (4)<br>No delivery cost estimated (5)
	CBA	Primary data collected (1)<br>Secondary national data from pilots, government and publications (2)<br>Assumptions by authors (3)<br>No delivery cost estimated (2)
Influenza	CEA	Secondary national data from pilots, government and publications (9)<br>Assumptions by authors (2)<br>No delivery cost estimated (1)
	CBA	Secondary national data from pilots, government and publications (1)
	CA	Primary data collected (2)
Varicella	CEA	Primary data collected (1)<br>Secondary national data from pilots, government and publications (3)<br>Secondary international data (1)<br>Assumptions by authors (2)<br>No delivery cost estimated (3)
	CBA	Secondary national data from pilots, government and publications (1)<br>No delivery cost estimated (1)
Meningococcal	CEA	Secondary national data from pilots, government and publications (3)<br>Assumptions by authors (4)<br>No delivery cost estimated (1)
	CA	Secondary national data from pilots, government and publication (1)
Measles	CEA	Primary data collected (1)<br>Secondary national data from pilots, government and publications (3)<br>Secondary international data (2)
	CBA	No delivery cost estimated (1)
Hepatitis B	CEA	Secondary national data from pilots, government and publications (3)<br>Secondary international data (2)
	CBA	No delivery cost estimated (1)
Typhoid	CEA	Secondary national data from pilots, government and publications (3)<br>Secondary international data (1)
	CA	Primary data collected (1)

**Vaccine**	**Type of cost analysis**	**Cost data source (number of studies)**
Oral cholera	CEA<br><br>CBA	Secondary national data from pilots, government and publications (1)<br>Secondary international data (2)<br>Primary data collected (1)
Polio	CEA<br><br>CBA	Secondary national data from pilots, government and publications (2)<br>No delivery cost estimated (1)<br>Secondary international data (1)
COVID-19	CEA<br><br>CA	Secondary national data from pilots, government and publications (2)<br>Secondary international data (1)<br>Primary data collected (1)
Measles-rubella	CEA	Secondary national data from pilots, government and publications (2)
Rubella	CEA	No delivery cost estimated (2)
Yellow fever	CEA	Secondary national data from pilots, government and publications (1)
Pentavalent	CA	No delivery cost estimated (1)
**Total**	CEA<br><br><br><br><br>CBA<br><br><br><br><br>CA	Primary data collected (5)<br>Secondary national data from pilots, government and publications (68)<br>Secondary international data (22)<br>Assumptions by authors (24)<br>No delivery cost estimated (24)<br>Primary data collected (2)<br>Secondary national data from pilots, government and publications (4)<br>Secondary international data (2)<br>Assumptions by authors (4)<br>No delivery cost estimated (7)<br>Primary data collected (6)<br>Secondary national data from pilots, government and publications (2)<br>No delivery cost estimated (1)

CA = cost analysis, CBA = cost-benefit analysis, CEA = cost-effectiveness analysis

Appendix 6. Figure on the studies by type of economic evaluation and completeness of vaccine delivery cost

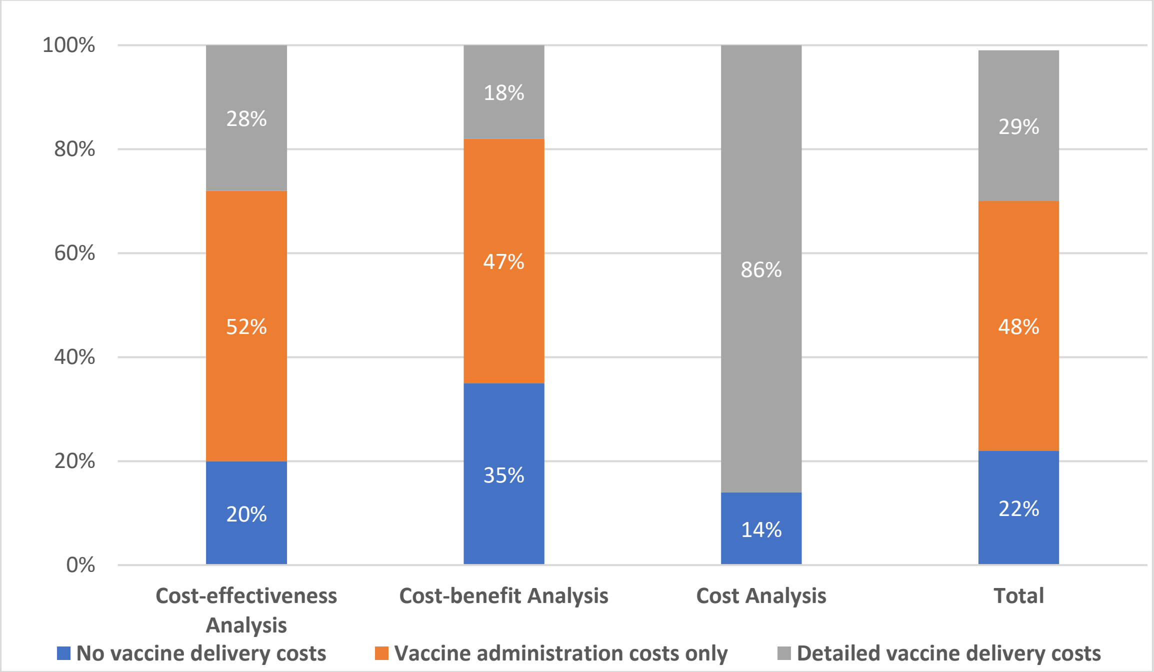

Appendix 7. Figure on common cost components of vaccine delivery costs included in studies with primary data collection

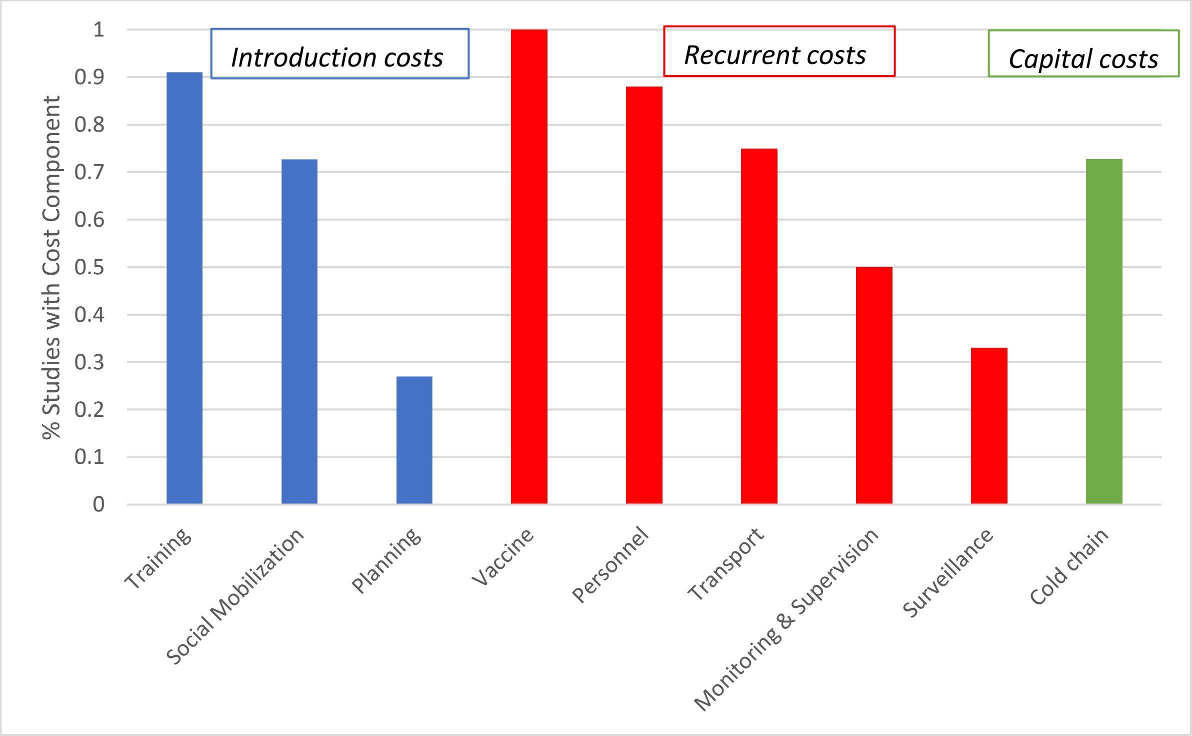

Appendix 8. Use cases for the new vaccine cost projection studies by vaccine

	Generate evidence on CEA or CBA for vaccine introduction to inform policymakers	To compare CEA of alternative vaccines	To compare CEA of alternative service delivery strategies	To estimate vaccine introduction cost for planning and advocacy	Total
Pneumococcal conjugate vaccine (PCV)	19	9	1	1	30
Human papillomavirus (HPV)	20	2	4	1	27
Rotavirus	19	5	0	1	25
Haemophilus influenzae type b (Hib)	14	1	2	0	17
Influenza	10	2	1	2	15
Varicella	10	1	1	0	12
Meningococcal	5	1	2	1	9
Measles	5	0	2	0	7
Hepatitis B	4	1	1	0	6
Typhoid	3	0	1	1	5
Oral cholera	3	0	1	0	4
Polio	1	3	0	0	4
COVID-19	3	0	1	0	4
Measles-rubella	1	1	0	0	2
Rubella	0	1	1	0	2
Yellow fever	1	0	0	0	1
Pentavalent	0	0	0	1	1
**Total**	118 (69%)	27 (16%)	18 (10%)	8 (5%)	171 (100%)
